# Inflammatory response and pneumocyte apoptosis during lung ischemia–reperfusion injury in an experimental pulmonary thromboembolism model

**DOI:** 10.1007/s11239-015-1182-x

**Published:** 2015-02-13

**Authors:** Chaosheng Deng, Zhenguo Zhai, Dawen Wu, Qichang Lin, Yuanhua Yang, Minxia Yang, Haibo Ding, Xiaoming Cao, Qiaoxian Zhang, Chen Wang

**Affiliations:** 1 Division of Respiratory & Critical Care Medicine, Scientific Research Department of the First Affiliated Hospital of Fujian Medical University, Fuzhou, 350005 Fujian People’s Republic of China; 2 Division of Respiratory & Critical Care Medicine, Affiliated Beijing Chaoyang Hospital of Capital University of Medical Science, Beijing, 100020 People’s Republic of China; 3 Division of Respiratory & Critical Care Medicine, First Affiliated Hospital of Fujian Medical University, Fuzhou, 35000 Fujian People’s Republic of China; 4 Division of Respiratory & Critical Care Medicine, Beijing Hospital of Ministry of Health, Beijing, 100020 People’s Republic of China

**Keywords:** Lung ischemia–reperfusion injury, Nitric oxide inhalation, Pulmonary thromboembolism, Inflammatory response, Pneumocyte apoptosis

## Abstract

Lung ischemia–reperfusion injury (LIRI) may occur in the region of the affected lung after reperfusion therapy. The inflammatory response mechanisms related to LIRI in pulmonary thromboembolism (PTE), especially in chronic PTE, need to be studied further. In a PTE model, inflammatory response and apoptosis may occur during LIRI and nitric oxide (NO) inhalation may alleviate the inflammatory response and apoptosis of pneumocytes during LIRI. A PTE canine model was established through blood clot embolism to the right lower lobar pulmonary artery. Two weeks later, we performed embolectomy with reperfusion to examine the LIRI changes among different groups. In particular, the ratio of arterial oxygen partial pressure to fractional inspired oxygen (PaO_2_/FiO_2_), serum concentrations of tumor necrosis factor-α (TNF-α), myeloperoxidase concentrations in lung homogenates, alveolar polymorphonuclear neutrophils (PMNs), lobar lung wet to dry ratio (W/D ratio), apoptotic pneumocytes, and lung sample ultrastructure were assessed. The PaO_2_/FiO_2_ in the NO inhalation group increased significantly when compared with the reperfusion group 4 and 6 h after reperfusion (368.83 ± 55.29 vs. 287.90 ± 54.84 mmHg, *P* < 0.05 and 380.63 ± 56.83 vs. 292.83 ± 6 0.34 mmHg, *P* < 0.05, respectively). In the NO inhalation group, TNF-α concentrations and alveolar PMN infiltration were significantly decreased as compared with those of the reperfusion group, 6 h after reperfusion (7.28 ± 1.49 vs. 8.90 ± 1.43 pg/mL, *P* < 0.05 and [(19 ± 6)/10 high power field (HPF) vs. (31 ± 11)/10 HPF, *P* < 0.05, respectively]. The amount of apoptotic pneumocytes in the lower lobar lung was negatively correlated with the arterial blood PaO_2_/FiO_2_, presented a positive correlation trend with the W/D ratio of the lower lobar lung, and a positive correlation with alveolar PMN in the reperfusion group and NO inhalation group. NO provided at 20 ppm for 6 h significantly alleviated LIRI in the PTE model. Our data indicate that, during LIRI, an obvious inflammatory response and apoptosis occur in our PTE model and NO inhalation may be useful in treating LIRI by alleviating the inflammatory response and pneumocyte apoptosis. This potential application warrants further investigation.

## Introduction

Currently, pulmonary thromboembolism (PTE) is the third most common cause of death in hospitalized patients [1]. After PTE treatment such as thrombolytic therapy, pulmonary embolectomy, pulmonary suction thrombectomy [2–4], or alternative interventional strategy of balloon pulmonary angioplasty for chronic thromboembolic pulmonary hypertension (CTEPH) [5], lung ischemia–reperfusion injury (LIRI) may occur in the region of the affected lung.

Because of the organization and recanalization channels within the chronic thrombus, the ischemic changes of chronic PTE may be reduced when compared to those brought about by deliberate/experimental ligation. In addition, the reperfusion pulmonary edema (RPE), which is one of the characteristics of LIRI, often develops within 48 h post-surgery such as thromboendarterectomy and lung transplant, thereby prolonging the intubation for mechanical ventilation, ventilator-associated infections, longer stays in the intensive care unit, and early postoperative mortality [6–8].

The inflammatory response plays a pivotal role in the development of LIRI after transplantation [9]. However, the inflammatory response mechanisms related to the LIRI in PTE, especially chronic PTE, are not studied as deeply as those involved in lung transplantation because there is no ideal PTE animal model due to the fact that animals do not develop spontaneous deep vein thrombosis or PTE.

With the guidance of Swan-Ganz float catheter under X-ray fluoroscopy, we have successfully established a reproducible modified experimental canine PTE model. This model mimics the pathological changes of chronic PTE and the location of thrombus is similar to that of CTEPH proximal type [10]. The pulmonary lower lobar artery is commonly involved due to a more extensive circulation in PTE [11]. Therefore, in this study, we aimed at precisely embolizing the right lower pulmonary lobar artery. Two weeks after the selective embolization, we performed the embolectomy with reperfusion to examine the LIRI changes, especially the inflammatory response during LIRI, in the canine PTE model.

## Materials and methods

### Animals and study design

Animal procedures were approved by the Fujian Medical University Institutional Animal Care and Use committee, and all experiments were conducted in strict accordance with the Guide for the Care and Use of Laboratory Animals.

Twenty-four healthy mongrel dogs (weight 20 ± 1.7 kg) were randomly divided into four groups. In the sham group (Group 1, *n* = 6), the procedures were the same as those performed in the other groups, except that 0.9 % NaCl was infused into the canine right lower pulmonary lobar artery to replace the autologous cylinder blood clots. The remaining 18 dogs underwent selective embolization. Twenty milliliters of autologous blood extracted from the dogs’ saphenous veins using a 20-mL syringe was rapidly injected into three segmental 7-cm cylinder tubes of pliable medical sterile intravenous transfusion polyvinyl chloride (PVC) tube with an inner diameter of 4 mm (the tube was named as tube I) to form the cylinder autologous blood clots at room temperature. Eight hours later, all blood clots were placed into a sterile container with 37 °C saline for later use. The right external jugular vein was then dissected and cannulated with a 7F sheath. A Swan-Ganz float catheter (Edwards Lifesciences Llc, Irvine, CA, USA) was used to guide another PVC tube, with a length of 40 cm and inner lumen diameter of 5 mm (tube II), to float selectively into the right lower pulmonary artery under X-ray fluoroscopic monitoring. The Swan-Ganz catheter was then extracted out from the inside of tube II and the three segmental autologous cylinder blood clots induced ex vivo were selectively injected into the right lower pulmonary lobar artery through the PVC tube II lumen. Later, oral, enteric-coated indomethacin tablets (0.5 mg/kg, 3 times/day for 3 days) were provided for pain relief and oral tranexamic acid (TXA) (110 mg/kg, every 12 h, for the duration of the experiment) was provided to inhibit endogenous fibrinolysis. Prophylactic penicillin (80,000 U/kg, twice daily for 1 week) was also provided to prevent infection. Two weeks later, the 18 dogs were subdivided into 3 groups. The ischemia group (Group 2, *n* = 6) underwent the same surgical procedures as the other groups, except the embolectomy, and the dogs were observed for 6 h. The reperfusion group (Group 3, *n* = 6) underwent embolectomy with reperfusion in the right lower lobar artery and the dogs were observed for 6 h after embolectomy. The NO inhalation group (Group 4, *n* = 6) underwent a process similar to the reperfusion group, but with additional NO inhalation at 20 ppm for 6 h through mechanical ventilation.

### Experimental PTE canine models and embolectomy

#### Preparing the animal model before embolectomy

Two weeks after embolization, the PTE dogs were anesthetized with 5 mL of intravenous propofol and intraperitoneal injection of 0.5 mL/kg of 3 % sodium pentobarbital. After endotracheal intubation, they were subsequently connected to Servo900C (SIEMENS, Bad Neustadt an der Saale, Germany) with volume controlled ventilation, tidal volume of 15 mL/kg, inspired oxygen concentration of 40 %, respiratory rates of 20 breaths/min, inspiratory time of 25 %, inspiratory pause time of 10 %, and positive end-expiratory pressure of 3 cm H_2_O. The arterial blood from the left femoral artery was periodically collected for analysis of the ratio of arterial oxygen partial pressure to fractional inspired oxygen (PaO_2_/FiO_2_).

#### Embolectomy with reperfusion and mechanical ventilation

A right thoracotomy was performed through the fifth intercostal space. The right lower pulmonary lobe was mobilized by dividing the pulmonary ligament and the hilar structures were then dissected. By clamping the right lower pulmonary artery hilum, Fogarty arterial embolectomy was performed according to the exact location of the thrombus where we had previously injected the clots through the PVC tube II lumen as described above. Anastomosis of the lower pulmonary artery was performed with non-absorbable 5–0 running sutures. The lower pulmonary artery was then unclamped and reperfusion changes were observed for 6 h.

In the NO inhalation group NO inhalation group, an NO delivery device (SensorNOx, SensorMedics Co. Yorba Linda, CA, USA) was introduced downstream of the humidifier through the inspiratory limb of the respiratory circuit after the embolectomy. NO was administered at a concentration of 20 ppm, starting immediately after initiating reperfusion and continuing for 6 h during the reperfusion period. The concentrations of NO and NO_2_ were determined continuously by the SensorNO_x_ delivery device, using electrochemical cell analysis. NO_2_ levels did not exceed 3 ppm.

A chest tube was inserted and the thoracotomy closed. Intravenous injection of 100 U/kg heparin was performed after every surgical procedure. Arterial PaO_2_/FiO_2_ was measured at baseline (0 h) and at 2, 4, and 6 h after surgical procedures. Each animal was covered during the experimental period to prevent hypothermia.

#### Treatment of the animals and lung tissues

The lung was removed from each animal for observation. Serum concentrations of tumor necrosis factor-α (TNF-α) were measured at different times. Alveolar polymorphonuclear neutrophils (PMNs) and myeloperoxidase (MPO) concentrations in lung homogenates were measured. The wet to dry ratio (W/D) of small fresh tissue in the segment part of the right lower lobe distal to the clot was measured and calculated. Lung tissue pathology, apoptotic pneumocytes, and ultrastructure were determined.

##### Serum TNF-α and MPO concentrations in lung homogenates

Serum TNF-α concentration at different times was measured by using an enzyme-linked immunosorbent assay (ELISA) kit (Medical Science And Technology Co., Ltd, Nuoshi, Beijing, China) according to the manufacturer’s instructions. The right lower lobe lung tissues were harvested, immediately weighed, and homogenized on ice in ten times their volume of normal saline. The homogenates were centrifuged at 3,000 rpm for 15 min. MPO levels in the supernatant were measured by using an ELISA kit (Assay Designs Inc., Ann Arbor, MI, USA) according to the manufacturer’s instructions.

##### Alveolar PMNs

Alveolar PMNs in the left lower lobar lung were observed under optical microscopy. Small pieces of the right lower lobe lung tissue were placed in 10 % formalin (Pharmaceutical company, Nai Ming, Shanghai, China), fixed, and paraffin embedded. Paraffin tissue sections were dewaxed, rehydrated, and stained with hematoxylin and eosin (H&E). After H&E staining, PMNs were counted in 10 continuous microscopic fields (magnification 400×) that only contained alveoli.

##### Lung W/D ratio

The right lower lobe lung tissue was excised, weighed immediately with a weighing scale (precision of 0.001 g), and then dried at 80 °C with continuous blowing for 72 h. The residuum was weighed and the W/D ratio was calculated.

##### H&E staining

The formalin-fixed lung tissues were embedded in paraffin and then cut into 4-mm-thick tissue sections, which were H&E stained.

##### Apoptotic pneumocytes

Terminal deoxynucleotidyl transferase dUTP nick end labeling (TUNEL) was performed according to the manufacturer’s instructions (R&D Systems, Minneapolis, MN, USA) for apoptosis detection. The slides were analyzed by a blinded pulmonary pathologist.

TUNEL-positive pneumocytes were counted in 100 microscopic fields (magnification 400×) per lung lobe. Only the cells lining the alveolar wall with positive nuclear and no cytoplasmic staining were regarded as apoptotic pneumocytes. Those found within the interstitium or in the alveoli were not counted.

##### Ultrastructure

Fresh lung tissue was cut into small pieces and immersed immediately in universal fixative (1 % glutaraldehyde, 4 % paraformaldehyde, pH 7.4), post-fixed in 2 % osmium tetroxide, dehydrated in graded acetones, and embedded in an Epon-Araldite mixture (Fisher Scientific Corp., Toronto, Canada). Selected blocks were thin-sectioned, mounted on copper grids, and contrasted with uranyl acetate and lead citrate. The grids were examined for pneumocytes using a Philips 208s electron microscope (N.V. Philips, Eindhoven, Netherlands).

### Statistical analysis

SPSS 11.0 software (IBM, Chicago, IL, USA) was used for statistical analysis. Numerical parameters with normal Gaussian distribution (According to the Kolmogorov–Smirnov test) are expressed as mean ± standard deviation. The difference of measured parameters between the different time points after surgery within the same group was analyzed by repeated-measures analysis of variance (ANOVA) and the differences between groups were assessed by ANOVA. Pearson’s correlation coefficient was used to assess the correlation between two variables. *P* < 0.05 was considered significant.

## Results

### A reddish brown thrombus was observed after embolectomy of the lower pulmonary lobar artery

**Fig. 1:**
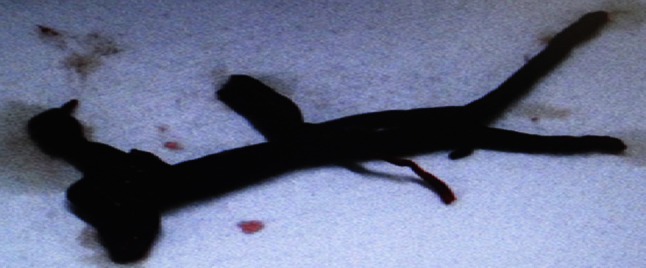
The thrombus was shown after embolectomy of the lower pulmonary lobar artery. Thrombi taken out from the lower pulmonary lobar artery after embolectomy were complete elongated strips with multiple pink granulation-like protrusions and multiple branches consistent with the pulmonary artery branches

Thrombi taken out from the lower pulmonary lobar artery after embolectomy were completely elongated strips with multiple pink granulation-like protrusions and multiple branches consistent with the pulmonary artery branches (Fig. 1).

### PaO_2_/FiO_2_ parameters at different time points

In the sham and ischemia groups, no significant difference was observed for the PaO_2_/FiO_2_ at different time points after surgical procedures (*P* > 0.05). The PaO_2_/FiO_2_ decreased significantly 2, 4, and 6 h after reperfusion when compared to the baseline. It was more obvious 4 h after reperfusion (287.90 ± 54.84 vs. 410.40 ± 28.36 mmHg, *P* < 0.05). In the NO inhalation group, PaO_2_/FiO_2_ decreased significantly 2 and 4 h after reperfusion compared to the baseline. It was more obvious 4 h after reperfusion (368.83 ± 55.29 vs. 441.43 ± 24.26 mmHg, *P* < 0.05) and increased gradually 6 h after reperfusion without a significant difference when compared with the baseline (380.63 ± 56.83 vs. 441.43 ± 24.26 mmHg, *P* > 0.05). PaO_2_/FiO_2_ in the NO inhalation group increased significantly when compared with the reperfusion group 4 and 6 h after reperfusion (368.83 ± 55.29 vs. 287.90 ± 54.84 mmHg, *P* < 0.05 and 380.63 ± 56.83 vs. 292.83 ± 60.34 mmHg, *P* < 0.05, respectively) (Table 1).

**Table 1:** Arterial blood PaO_2_/FiO_2_ at each time point after surgical procedures (mean ± standard deviation)

	0 h	2 h	4 h	6 h
PaO_2_/FiO_2_ (mmHg)				
Group 1	484.42 ± 28.34	475.50 ± 20.80	543.45 ± 61.56	423.20 ± 23.20
Group 2	430.95 ± 28.37	428.75 ± 37.50	431.25 ± 39.24	435.00 ± 31.62
Group 3	410.40 ± 28.36	335.55 ± 29.29	287.90 ± 54.84*	292.83 ± 60.34
Group 4	441.43 ± 24.26	371.87 ± 20.35	368.83 ± 55.29^#,◆^	380.63 ± 56.83^#,◆^

*Group 1* Sham group, *Group 2* ischemia group, *Group 3* reperfusion group, *Group 4* NO inhalation group

^*,^^#^ *P* < 0.05 compared with the parameters recorded at the time point before reperfusion within the same group

^◆^ *P* < 0.05 at the same time point among different groups, compared with the reperfusion group

### Inflammatory response during LIRI in the PTE model

#### Serum TNF-α concentrations

**Fig. 2:**
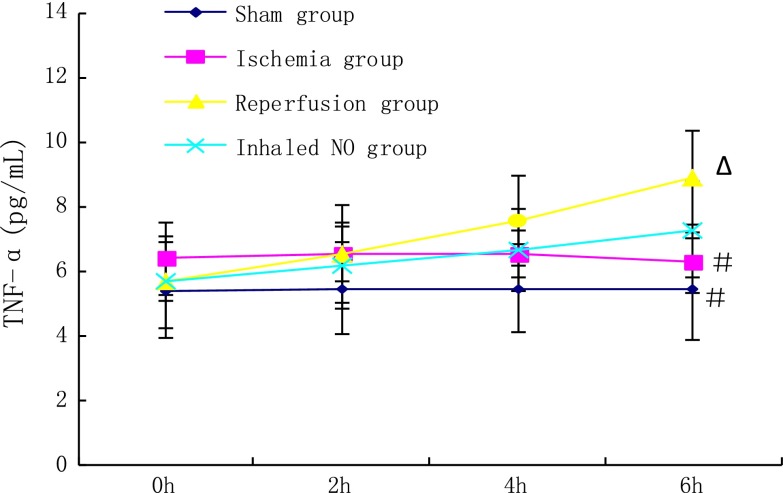
Serum TNF-α concentrations at each time point after surgical procedures. Statistical analysis was performed at the same time point, 6 h after reperfusion, among different groups. ^#^ *P* < 0.05 compared with the reperfusion group, ^Δ^ *P* < 0.05 compared to the NO inhalation group

Serum TNF-α concentrations in the reperfusion group increased significantly 6 h after reperfusion as compared with the baseline value and the value at 2 h after reperfusion (8.90 ± 1.43 vs. 5.67 ± 1.43 pg/mL, *P* < 0.05 and 8.90 ± 1.43 vs. 6.54 ± 1.53 pg/mL, *P* < 0.05, respectively) (Fig. 2). TNF-α concentration in the reperfusion group was much higher than that of the ischemia and sham groups (8.90 ± 1.43 vs. 6.28 ± 0.94 pg/mL, *P* < 0.05 and 8.90 ± 1.43 vs. 5.44 ± 1.58 pg/mL, *P* < 0.05, respectively) (Fig. 2). In the NO inhalation group, TNF-α concentration was significantly decreased compared to that of the reperfusion group (7.28 ± 1.49 vs. 8.90 ± 1.43 pg/mL, *P* < 0.05) (Fig. 2).

#### Alveolar PMN infiltration and MPO concentration in lung homogenates

**Fig. 3:**
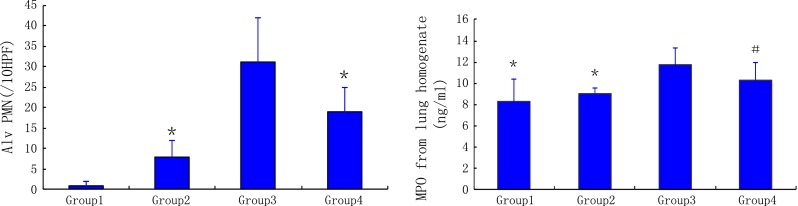
Alveolar PMN and MPO concentration in lung homogenates after surgical procedures. *Group 1* sham group, *Group 2* ischemia group, *Group 3* reperfusion group, *Group 4* NO inhalation group. ^*,^^#^ *P* < 0.05 compared with the reperfusion group

Alveolar PMN infiltration in the NO inhalation group significantly decreased compared to that of the reperfusion group (19 ± 6)/10 high power field (HPF) vs. (31 ± 11)/10 HPF, *P* < 0.05) (Fig. 3-1). Alveolar PMN infiltration in the reperfusion group was much higher than that of the ischemia group ((31 ± 11)/10 HPF vs. (8 ± 4)/10 HPF, *P* < 0.05) (Fig. 3-1). MPO concentrations in lung homogenates from the reperfusion group were much higher than those of the NO inhalation group, ischemia, and sham groups (11.74 ± 1.63 vs. 10.29 ± 1.70 ng/mL, *P* < 0.05, 11.74 ± 1.63 vs. 9.08 ± 0.46 ng/mL, *P* < 0.05 and 11.74 ± 1.63 vs. 8.30 ± 2.13 ng/mL, *P* < 0.05, respectively) (Fig. 3-2).

#### Lung sample ultrastructure evaluated by electron microscopy

**Fig. 4:**
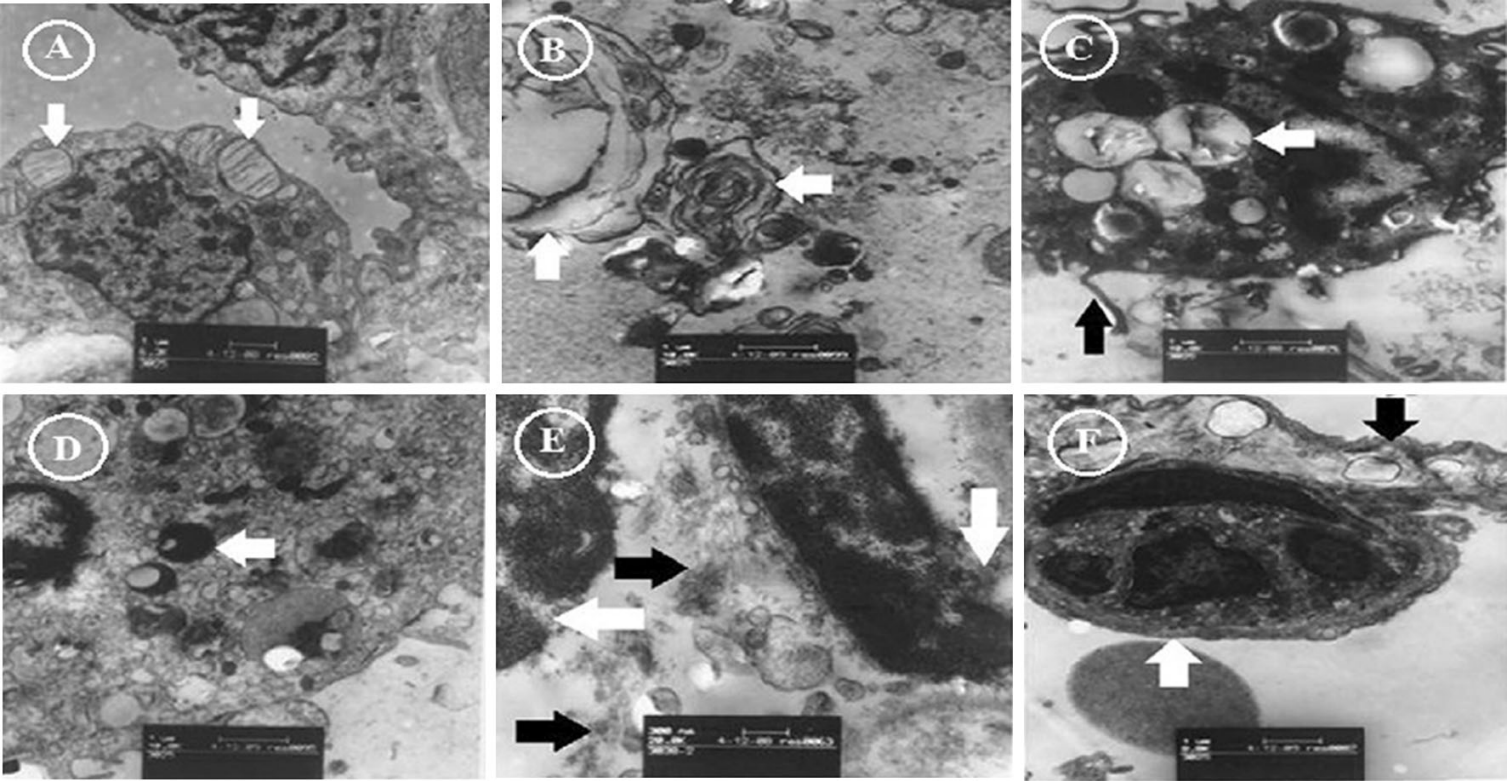
The lung specimen ultrastructures were evaluated by electron microscopy. **a** In the sham group, lung ultrastructural architecture was normal with type II pneumocytes containing normal lamellar bodies (*white arrow*). **b** Swelling of lamellar bodies (*white arrow*) in some type II pneumocytes were observed in the reperfusion group. **c** Long pseudopodia (*black arrow*) of macrophage phagocyting swelling and lamellar bodies with vacuoles degeneration (*white arrow*) in the reperfusion group. **d** Highly electronic intensity dot in macrophages after phagocytosis of necrotic tissue (*white arrow*). **e** Disintegrated naked nuclei (*white arrow*) and other structure of the amorphous necrotic material (*black arrow*) were observed in the alveolar cavity in the reperfusion group. **f** PMNs (*white arrow*) were in close contact with alveolar epithelial cells with vacuoles degeneration (*black arrow*) in the reperfusion group

In the sham group, lung ultrastructural architecture was normal and type II pneumocytes with lamellar bodies were observed (Fig. 4a). Swelling of lamellar bodies in some type II pneumocytes were detected in the reperfusion group (Fig. 4b). Macrophages with long pseudopodia, phagocyting swelling, and vacuoles degeneration of lamellar bodies were observed in the reperfusion group (Fig. 4c). High electronic intensity dots were phagocytosed by macrophages (Fig. 4d). Disintegrated naked nuclei and other amorphous necrotic material were observed in the alveolar cavity in the reperfusion group (Fig. 4e). PMNs were in close contact with alveolar epithelial cells with vacuole degeneration in the reperfusion group (Fig. 4f).

### W/D ratio of the right lower lobar lung

**Fig. 5:**
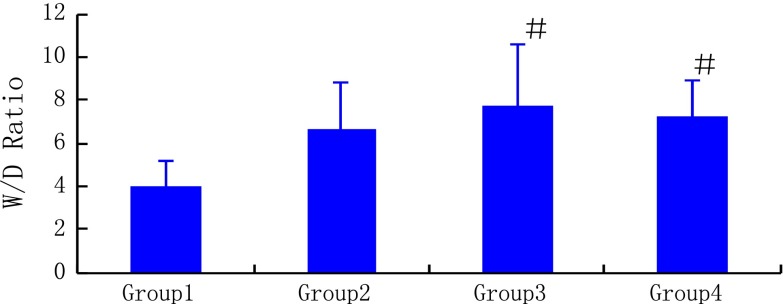
Lung W/D ratio after surgical procedures. *Group 1* sham group, *Group 2* ischemia group, *Group 3* reperfusion group, *Group 4* NO inhalation group. ^#^ *P* < 0.05 compared with the sham group

The right lower lobe W/D ratios in the reperfusion and NO inhalation groups were significantly higher than that of the sham group (7.73 ± 2.81 vs. 4.02 ± 1.13, *P* < 0.05 and 7.23 ± 1.67 vs. 4.02 ± 1.13, *P* < 0.05, respectively) (Fig. 5).

### H&E staining (400×)

**Fig. 6:**
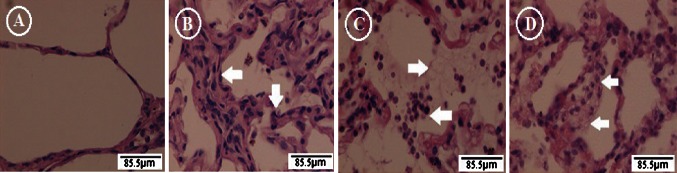
Lung tissue pathology at each time point after surgical procedures (H&E ×400). **a** The alveolar structure in the right lower lung from a sham animal was shown. **b** Some collapsed alveolar structures (*white arrow*), thickened alveolar septa (*white arrow*), and a few exudative cells in the alveolar space were observed in the ischemia group. **c** Incomplete and destructed alveolar structure with a large number of exudative cells, mainly PMNs, and exudation (*white arrow*) were observed within the alveolar spaces in the reperfusion group. **d** Similar incomplete and destructed alveolar structures with exudative cells, mainly PMNs, and exudation (*white arrow*) were observed in the NO inhalation group, but to a lesser extent than that observed in the reperfusion group

The alveolar structure with some exudation in the right lower lung of the sham group is shown (Fig. 6a). Some collapsed alveolar structures, thickened alveolar septa, and a few exudative cells in the alveolar space were observed in specimens from the ischemia group (Fig. 6b). Incomplete and destructed alveolar structure with a large number of exudative cells, mainly PMNs, and exudation were detected within the alveolar space in the reperfusion group (Fig. 6c). Similar incomplete and destructed alveolar structures with exudative cells, mainly PMNs, and exudation were observed in the NO inhalation group, but to a much lower degree than observed in the reperfusion group (Fig. 6d).

### Apoptotic pneumocytes among the different groups

**Fig. 7:**
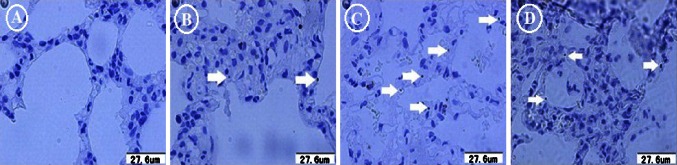
Apoptotic pneumocytes detected by TUNEL. **a** No apoptotic cell was detected in the sham group. **b** Two apoptotic pneumocytes were detected (*white arrow*) in the ischemia group. **c** Six hours after surgery, five apoptotic pneumocytes were observed (*white arrow*) in the reperfusion group. **d** Three apoptotic pneumocytes were detected (*white arrow*) in the NO inhalation group

Apoptotic pneumocytes were observed in the segment distal from the clot. No apoptotic cell was detected in the sham group (Fig. 7a). In the ischemia group, some apoptotic pneumocytes (2 ± 1 pneumocytes/5 fields, 400×) were revealed by using TUNEL (Fig. 7b). Six hours after surgery, the number of apoptotic pneumocytes in the reperfusion group increased significantly (5 ± 1 pneumocytes/5 fields) (Fig. 7c). In the NO inhalation group, the number of apoptotic pneumocytes decreased (3 ± 1 pneumocytes/5 fields) (Fig. 7d).

### Correlation between the amount of apoptotic pneumocytes and PaO_2_/FiO_2_, W/D ratio of the lower lobar lung, and alveolar PMNs

In the reperfusion and NO inhalation groups, the amount of apoptotic pneumocytes in the lower lobar lung was negatively correlated with the arterial blood PaO_2_/FiO_2_ (*r* = −0.74, *P* < 0.05 and *r* = −0.80, *P* < 0.05, respectively),presented a trend toward a positive correlation with the W/D ratio of the lower lobar lung (*r* = 0.6, *P* > 0.05 and *r* = 0.5, *P* > 0.05, respectively), and was positively correlated with alveolar PMNs (*r* = 0.84, *P* < 0.05 and *r* = 0.92, *P* < 0.05, respectively) (Table 2).

**Table 2:** Pearson correlation coefficient (r) between the number of apoptotic pneumocytes and arterial blood PaO_2_/FiO_2_, lower lobar lung W/D ratio, and alveolar PMNs

		Reperfusion group			NO inhalation group		
		PaO_2_/FiO_2_	W/D ratio	PMNs	PaO_2_/FiO_2_	W/D ratio	PMNs
Number of apoptotic pneumocytes	*r*	−0.74	0.6	0.84	−0.80	0.5	0.92
*P* value		0.04	0.09	0.04	0.02	0.12	0.02

### Correlation between the amount of alveolar PMNs and arterial blood PaO_2_/FiO_2_, lower lobar lung W/D ratio, MPO, and TNF-α concentrations

The amount of alveolar PMNs in the lower lobar lung was negatively correlated with the arterial blood PaO_2_/FiO_2_ (*r* = −0.97, *P* < 0.05). A positive correlation trend was observed with the W/D ratio of the lower lobar lung (*r* = 0.86, *P* > 0.05). A positive correlation with MPO concentrations in lung homogenates (*r* = 0.98, *P* < 0.05) and serum TNF-α concentration (*r* = 0.99, *P* < 0.05) was also observed (Table 3).

**Table 3:** Pearson correlation coefficient (r) between the number of alveolar PMNs and arterial blood PaO_2_/FiO_2_, lower lobar lung W/D ratio, MPO concentration, and TNF-α concentration

		PaO_2_/FiO_2_	W/D ratio	MPO	TNF-α
Alveolar PMNs	*r*	−0.97	0.86	0.98	0.99
*P* value		0.03	0.14	0.012	0.003

## Discussion

### A modified canine PTE model

We have successfully established a modified canine PTE model. This model mimics PTE pathological changes. A precise embolization into the intended right lower pulmonary artery enabled us to perform embolectomy to investigate the inflammatory response to LIRI in the PTE model.

In the PTE model, thrombi taken out from the lower pulmonary lobar artery after embolectomy were complete elongated strips with multiple pink granulation-like protrusions and multiple branches consistent with the pulmonary artery branches.

### Ischemia–reperfusion injury

In general, vascular response includes at least two phases, ischemia and reperfusion, resulting in ischemia–reperfusion injury in systemic vascular beds. As demonstrated by H&E staining in our ischemia group, LIRI results in a moderate inflammation characterized by a ‘chronic’ fibroproliferative state, including infiltration and pulmonary remodeling [12]. In the ischemic phase, the unresolved clot and inflammatory cells provide the microenvironment and may stimulate cell proliferation and injury to the cells, which is associated with lack of oxygen and cell damage [13, 14].

Once the blood flow is re-established, the injury elicited by reperfusion can be more severe than that caused by ischemia per se. The injury may also be attributed to the combined effects of ischemia and reperfusion [15]. RPE is the most important factor complicating the early postoperative period after pulmonary thromboendarterectomy [16]. In our experimental model, an incompletely destructed alveolar structure with a large number of exudative cells and exudation was observed within the alveolar spaces as revealed by H&E staining after embolectomy of the lobar artery. Therefore, the significant increase in W/D ratio and alveolar PMNs may impair gas exchange (PaO_2_/FiO_2_ decreased) due to increased edema formation and surfactant inactivation as often occurs in patients after endarterectomy [17].

#### Injury mechanisms during LIRI

LIRI is due primarily to mechanisms that cause alveolar–capillary barrier, especially alveolar epithelial cell damage, and that increase pulmonary vascular permeability, which is associated with the formation of reactive oxygen intermediates, endothelial cell injury, cytokine activity, neutrophil activation, complement activation, and inflammation response [18, 19].

##### Reactive oxygen species (ROS) generation

It is commonly believed that reperfusion lung injury is primarily based on the generation of ROS on reperfusion, which may cause cellular damage and apoptosis [20, 21]. In hypoxic conditions, the inducible nitric oxide synthetase (iNOS) can be enhanced, which may lead to ROS-type cellular injury and increase oxidant radical byproducts, including the peroxynitrite anion [22]. ROS have diverse actions on pulmonary tissue, including cell proliferation, gene transcription, smooth muscle contraction, and interaction with redox enzymes [23]. The array of inflammatory mediators released into the circulation, in turn, governs the chemoattraction of various nonresident leukocytes that initiate the production of ROS [24]. Of the chemoattracted cell types, neutrophils and monocytes possess the greatest ROS-generating potential [25]. Therefore, during LIRI, the damage and apoptosis in the lung may be more serious.

##### The inflammatory response in our LIRI model

The inflammatory response plays a pivotal role in the development of LIRI after transplantation. This inflammatory process is characterized by the infiltration of PMNs and other inflammatory cells such as macrophages that release inflammatory mediators, including TNF-α.

*Roles of macrophages and TNF-*α *during LIRI in the PTE model* Macrophages play a key role in the response to LIRI [26]. They are known as “the defenders of human health” by phagocyting bacteria, viruses, foreign bodies, damaged cells, and necrotic tissue. In our study, the lung ultrastructural architecture showed that swelling of lamellar bodies occurred in some type II pneumocytes in the reperfusion group. Additionally, macrophages with long pseudopodia phagocytosed the swelling and vacuolar lamellar bodies. Moreover, disintegrated naked nuclei and other structures of the amorphous necrosis material were also observed in the alveolar cavity.

Macrophages may also promote the production of inflammatory mediators [26]. Damaged cells spill cytoplasmic and nuclear components into the extracellular milieu, which activate macrophages, leading to the production of pro-inflammatory cytokines and chemokines, including TNF-α [27]. TNF-α is a pro-inflammatory factor that is released by lung macrophages in early stages and plays an important role in initiating lung inflammation [28]. Another study showed that there was a close correlation between pro-inflammatory factors and LIRI in rabbit models after the left pulmonary artery occlusion was released. TNF-α level was continuously elevated in the reperfusion group [29]. In our experimental model, serum TNF-α concentrations in the reperfusion group increased significantly 6 h after reperfusion as compared with the baseline value and the value at 2 h after reperfusion. TNF-α concentrations were also much higher than those of the ischemia and sham groups. TNF-α released by macrophage is an objective index to evaluate the severity of inflammatory response during LIRI in PTE. Therefore, we can attenuate acute LIRI by reducing TNF-α level [29]. TNF-α produced by macrophages can also lead to the recruitment of PMNs to the injured tissue [27], which may be responsible for serious lung damage during LIRI in PTE.

*Roles of PMN and MPO during LIRI in the PTE model* After ischemia–reperfusion, PMN infiltration into the alveolar cavity increased significantly and PMNs are responsible for tissue damage [30]. In our experimental model, alveolar PMN infiltration in the reperfusion group was much higher than that of the ischemia group, resulting in the destruction of alveolar structure as observed by H&E staining, including an incompletely destructed alveolar structure with a large number of exudative cells and exudation. MPO concentrations in the lung tissue are related to neutrophil activation [31]. In this study, MPO concentrations from lung homogenates in the reperfusion group were much higher than those of the ischemia and sham groups. Meanwhile, the amount of alveolar PMNs in the lower lobar lung was positively correlated with the lung MPO, indicating PMN recruitment and progressive activation in the lung tissue. One study demonstrated that PMNs play an important role in LIRI in a model of rat lung transplantation and that the gas exchange of the transplanted lung could be improved by reducing alveolar PMN infiltration [32]. In this study, PMN infiltration was also negatively correlated with the arterial blood PaO_2_/FiO_2_. Therefore, it is vital to control PMN activation to avoid excessive tissue damage during PTE reperfusion.

*Interaction between PMNs and macrophages during LIRI in the PTE model* The inflammatory mediators produced by macrophages can also stimulate the recruitment of neutrophils to the injured tissue. It has been shown in experimental models of inflammation and also in clinically relevant models that macrophage-derived chemokines (TNF-α) promote neutrophils’ egress from the vasculature [27]. In this study, electron microscopy showed that PMNs were in close contact with the alveolar epithelial cells with vacuoles degeneration in the reperfusion group, and the amount of alveolar PMNs in the lower lobar lung was positively correlated with serum TNF-α concentrations. Thus, PMN recruitment to the injured lung tissue may be due to the increased concentrations of TNF-α produced by macrophages during LIRI in the chronic PTE model.

*Apoptotic pneumocytes after ischemia–reperfusion* When a cell is sufficiently injured, cell death occurs either through necrosis or apoptosis. Apoptosis is morphologically characterized by nuclear condensation and shrinkage followed by fragmentation of nuclear chromatin without typical inflammation. Apoptosis was determined by TUNEL in this study. Studies indicated that RPE clinical manifestations after interventions for PTE are similar to those of lung transplantation [5–8]. A significant number of pneumocytes undergo apoptosis after reperfusion in the transplanted rat lung [33]. In our experimental model, the number of apoptotic pneumocytes increased after reperfusion similarly to our previous study [34]. The amount of apoptotic pneumocytes in the lower lobar lung was also negatively correlated with the arterial blood PaO_2_/FiO_2_ in the reperfusion and NO inhalation groups. Therefore, during LIRI in PTE, pneumocyte apoptosis may be attributed to the low PaO_2_/FiO_2_, which is similar to triggering apoptosis by exposure to certain environmental conditions such as hypoxic conditions [20] and may result from the increased number of alveolar PMNs after reperfusion.

### NO inhalation improved PaO_2_/FiO_2_, macrophage numbers and TNF-α levels, PMN numbers and MPO levels

NO inhalation may be useful to treat acute and chronic pulmonary embolism due to its vasodilatation property [35, 36]. In the NO inhalation group, the PaO_2_/FiO_2_ significantly decreased 2 and 4 h after reperfusion. However, due to the improvement in ratio of ventilation and blood flow (V/Q matching) [3, 37, 38], the PaO_2_/FiO_2_ increased gradually to the baseline 6 h after reperfusion. Compared with the reperfusion group, the PaO_2_/FiO_2_ increased significantly after inhalation of 20 ppm NO for 4 or 6 h, related to elevated inducible nitric oxide synthase (iNOS) expression and its activity [39].

After NO inhalation, LIRI can be effectively blunted by reduction of the macrophage-dependent injury and be attenuated by minimizing neutrophil sequestration [40]. In our study, in the NO inhalation group, TNF-α concentration decreased significantly when compared with that of the reperfusion group. Our results are in agreement with data indicating that breathing NO prevented the induction of TNF-α production and that NO inhalation improves outcomes after successful cardiopulmonary resuscitation in mice [41]. Alveolar PMN infiltration and MPO concentration in the lung homogenates in the NO inhalation group decreased significantly as compared with those of the reperfusion group, which may possibly result from the decreased production of macrophage-derived chemokines such as TNF-α. A study showed that a short period (10 min) of NO inhalation preconditioning with low concentration can alleviate LIRI in mice and that it is associated with the inhibition of toll-like receptor 2/4 in the lung after LIRI [42]. However, in our study, decreased TNF-α concentrations and alveolar PMN infiltration can be observed after 4–6 h of NO inhalation when compared with the reperfusion group. Hence, a moderate duration of NO inhalation can alleviate LIRI in PTE.

The therapeutic window for NO applications is narrow because NO inhalation can be either protective or toxic to the lung depending on the dose, timing, duration of NO administration, source of NO, and the local redox environment [43–45]. A study showed that the maximum protective effect is achieved with NO concentrations between 10 and 20 ppm [46]. NO inhalation is routinely provided for the first 4 h postoperatively at doses of 15–20 ppm [47].

### Limitations and clinical implications

The mechanisms of LIRI are complex and may include neutrophil activation, cytokines, ROS, arachidonic acid derivatives, complement, hemolysis, thromboxane/PGF2, and platelet activating factor, causing cellular damage and apoptosis. In addition, the inflammatory response includes various inflammatory cell infiltration and proinflammatory cytokine release. Therefore, further studies should focus intensively on the interactions between inflammatory response factors during LIRI in PTE. The routine use of NO inhalation after lung surgery for PTE should also be further studied.

## Conclusions

Our PTE model allowed us to observe an obvious inflammatory response and apoptosis during LIRI.

It seems that the pneumocyte damages caused by the inflammatory response are more serious than apoptosis during LIRI in PTE. Remarkably, physiological improvements are observed when NO inhalation is used as a therapeutic approach to treat LIRI in a canine PTE model.

NO inhalation may be useful in treating LIRI resulting from acute or chronic PTE by alleviating the inflammatory response and pneumocyte apoptosis. This potential application warrants further investigation.
